# Protective effects of *Elaeagnus angustifolia *L. fruit extract on CCl_4_-induced oxidative stress and inflammation in rats liver

**DOI:** 10.22038/ajp.2025.25915

**Published:** 2025

**Authors:** Masoud Ojarudi, Ali Golchin, Hamid Reza Karamdel, Mohammad Valilo, Parviz Ranjbarvan

**Affiliations:** 1 *Student Research Committee, Urmia University of Medical Sciences, Urmia, Iran*; 2 *Department of Biochemistry, Faculty of Medicine, Urmia University of Medical Sciences, Urmia, Iran*; 3 *Solid Tumor Research Center, Cellular and Molecular Medicine Research Institute, Urmia University of Medical Sciences, Urmia, Iran*; 4 *Department of Applied Cell Sciences, School of Medicine, Urmia University of Medical Sciences, Urmia, Islamic Republic of Iran*; 5 *Cellular and Molecular Research Center, Cellular and Molecular Medicine Research Institute, Urmia University of Medical Sciences, Urmia, Iran*

**Keywords:** Elaeagnus angustifolia L., Carbon tetrachloride, Liver damage, Oxidative stress, Antioxidant

## Abstract

**Objective:**

Hepatic cells face oxidative stress-induced damage, but plant antioxidants may offer protection. This study aimed to assess *Elaeagnus angustifolia *L. fruit extract's potential in shielding rat livers from CCl_4_ damage.

**Materials and Methods:**

30 Male Wistar rats were randomly divided into five groups: normal control (received distilled water), *E. angustifolia* hydroalcoholic extract control, CCl_4_ control, *E. angustifolia *extract pretreatment (600 mg/kg), and silymarin pretreatment (100 mg/kg). After 14 days of oral administration of extracts, CCl_4_ was injected intraperitoneally. The samples were collected 48 hr later. Histological and biochemical analyses were then carried out.

**Results:**

CCl_4_ injection caused significant (p<0.001) changes in liver serum enzymes, lipid profile, bilirubin, total protein, serum albumin, antioxidant enzymes, malondialdehyde, Inflammatory cytokines, and liver tissue morphology. *E. angustifolia *extract pre-treatment significantly (p<0.05) returned changes to the normal state.

**Conclusion:**

This study's findings revealed that *E. angustifolia *extract pretreatment could reduce liver injury caused by CCl_4_ in rats.

## Introduction

The liver is crucial for processing exogenous and endogenous compounds, making it susceptible to daily oxidative stress (Ahmadi et al. 2022; Yu et al. 2018). Excessive ROS (Reactive oxygen species) from various sources can damage nucleic acids, proteins, lipids, and membranes, affecting liver function despite antioxidant defenses (Ullah et al. 2020). Hence, investigating this field is imperative, and animal models can be utilized for studying liver damage induced by oxidative stress in research. 

CCl_4_ is a known inducer of oxidative liver damage in laboratory animals. CCl_4_ induces oxidative liver damage by forming radicals that bind to biomolecules, causing lipid peroxidation and membrane damage, exacerbating inflammation and injury (Rezagholizadeh et al. 2022).

Exploring natural sources for antioxidant compounds can be a promising approach to prevent oxidative damage. Medicinal plants provide antioxidants like polyphenols and flavonoids for protection (El-Yagoubi et al. 2024; Valvi et al. 2016). *Elaeagnus angustifolia *L. fruit is also rich in antioxidant compounds.

*Elaeagnus angustifolia *L.*, *a small tree or shrub, is rich in phytochemicals like terpenoids, flavonoids, tannins, and vitamins. Main flavonoids include catechin, epigallocatechin, and chlorogenic acid (Hassanzadeh and Hassanpour 2018).

Silymarin, derived from *Silybum marianum* and commonly referred to as milk thistle, is an extract that has been utilized for centuries in the treatment of liver diseases (Gillessen and Schmidt 2020). The flavonoid silymarin, along with its structural component silibinin, are compounds that have been shown to possess hepatoprotective properties (Fraschini et al. 2002). In most studies, silymarin is employed as a standard treatment for liver conditions (Barreiro Carpio et al. 2024; El Rabey et al. 2021).

Hence, based on the antioxidant properties found in the fruit of this plant, this study aimed to evaluate hepatoprotective effects of *E. angustifolia *L. fruits in the CCl_4_-induced rats.

## Materials and Methods

### Chemicals

Chemicals were sourced from Merck (Germany), including methanol, thiobarbituric acid, serum albumin, hydrogen peroxide, and more. Reagents like Folin-Ciocalteu, DPPH (2,2-diphenyl-1-picrylhydrazyl), gallic acid, and quercetin were from Sigma. Serum parameters were analyzed using kits from Pars-Peyvand Co., while SOD (Superoxide dismutase), GPx (Glutathione peroxidase), TNF-α (Tumor necrosis factor alpha), and IL-6 (Interleukin 6) assay kits were from Zell Bio and karmania pars gene Co, Kerman, Iran.

### Extraction method

Dried fruit of* E. angustifolia *was purchased from Iran Extract Research Institute (The genus and species were definitively identified by a botanist), ground, and macerated in 70% methanol (500 g of *E. angustifolia *fruit powders in 2 L of 70% methanol) for four days. The resulting macerate was filtered, dried at room temperature, freeze-dried for two days, and stored in a freezer (the maceration process yielded 80 g of crude *E. angustifolia *extract) (Ojarudi et al. 2020).

### Total phenolic, total flavonoid content, and DPPH radical scavenging activity

Total phenolic content was assessed using the Folin-Ciocalteo method, while total flavonoid content was determined through a colorimetric method similar to Kadhim et al (Jawad Kadhim et al. 2019). Antioxidant activity was measured using the DPPH radical scavenging method (Csicsor and Tombácz 2022).

### Animals and experimental procedure

The study utilized 30 male Wistar rats (200-220 g) housed in a temperature-controlled room with a 12-hour light-dark cycle. Animals were divided onto five different groups at random:

1- Normal Control: received distilled water orally + olive oil intraperitoneally

2- *E. angustifolia*: received *E. angustifolia *fruit extract orally (600 mg/kg) + olive oil intraperitoneally

3- CCl_4_: received distilled water orally + CCl_4_ intraperitoneally

4- *E. angustifolia* + CCl_4_: received *E. angustifolia* fruit extract orally (600 mg/kg) + CCl_4_ intraperitoneally

5- Silymarin + CCl_4_: received Silymarin orally (100 mg/kg (Ojarudi et al. 2020)) + CCl_4_ intraperitoneally

Considering the previous studies indicating that the optimal therapeutic dosage of *E. angustifolia* fruit extract is 600 mg/kg, this dosage was used in the current research (Dabbaghmanesh et al. 2017). Also, according to similar studies (Ojarudi et al. 2020), the pretreatment group receiving silymarin was used to compare the pretreatment effects of *E. angustifolia* extract.

All groups were treated for 14 days. On day 14, groups three, four, and five received 1 ml/kg of a CCl_4_ and olive oil mixture intraperitoneally, while groups one and two received only olive oil (the solvent of CCl_4_) (Mahmoodzadeh et al. 2017). After 48 hr and a 12-hr fast, animals were anesthetized (Intraperitoneally injection of 10 mg/kg ketamine and 90 mg/kg xylazine per body weight), blood and liver tissue samples were collected for analysis.

### Serum factor levels

Serum factors, including liver enzymes (AST, ALT, ALP, and GGT [Gamma glutamyl transferase]), total protein, albumin, lipid profile (total cholesterol, triglycerides, LDL-C, and HDL-C), and bilirubin, were analyzed using an autoanalyzer (Biochemistry Analyzer BT 3000, Italy) following the instructions provided in each laboratory kit.

### Protein assay

Tissue protein was measured using Bradford's method (Bradford 1976).

### MDA

The concentration of liver tissue MDA was determined using the Uchiyama & Mihara technique, with little modification similar to our earlier work (Mihara and Uchiyama 1978; Ojarudi et al. 2020).

### Total antioxidant capacity

The FRAP method was used to determine total antioxidant capacity (Benzie and Strain 1996).

### Antioxidant enzymes

The enzymatic activity of CAT (Catalase) present in the tissue was quantified utilizing the Aebi’s method (Aebi 1984). Furthermore, the activity of both SOD and GPx enzymes was evaluated using the Zell Bio kit's procedure.

### Inflammatory cytokines measurement

The levels of TNF-α and IL-6 were measured by the relevant commercial ELISA kit with the manufacturer's protocol.

### Histopathological study

Liver tissue samples were fixed in 10% formalin, dehydrated in ethanol (50-100%), cleared with Xylene, embedded in paraffin, sectioned (4-5 µm), and stained with hematoxylin-eosin.

### Liver index and weight

The body mass and liver mass of each rat were measured at the conclusion of the experiment. The liver index (Eidi et al. 2013b), which is the ratio of liver mass to body mass, was then computed using the following formula: 

Liver index = (liver weight/ body weight) × 100%

### Statistical analysis

Data is reported as mean ± standard deviation. Data analysis was done by one-way analysis of variance with GraphPad Prism 8.3.0. Post hoc comparisons were made using Tukey's test to determine specific group differences. A significance level of p<0.05 was considered. The experiments were conducted in triplicate to ensure the reliability and reproducibility of the results.

## Results

### Contents of total phenolic and total flavonoid and antioxidant activity

Table 1 displays the phenolic and flavonoid content as well as the *in vitro* antioxidant activity of the extract. Antioxidant activity is presented as 50% inhibition of free radicals (IC50), representing the extract concentration (µg/ml) needed to scavenge 50% of DPPH radicals.

### Effects of E. angustifolia extract administration on liver enzymes

CCl_4_ administration significantly increased liver enzyme levels (AST, ALT, ALP, and GGT) (p<0.001), which were attenuated by pretreatment with *E. angustifolia* extract (p<0.01), as shown in Table 2.

**Table 1:** Phenolic and flavonoid content and antioxidant activity of the Elaeagnus angustifolia L. fruit extract

DPPH scavenging activity IC_50_ value (µg/ml)	Total flavonoid content(mg QU/gram of extract)	Total phenolic content(mg GA/gram of extract)	Sample
**144.71**	23.81±0.71	36.04±0.49	*E. angustifolia*

GA: Gallic acid, QU: Quercetin

**Table 2:** The effect of Elaeagnus angustifolia L. fruit extract on liver enzymes

GGT (IU/L)	ALP (IU/L)	ALT (IU/L)	AST (IU/L)	Groups
**1.42±0.29**	429.16±6.55	48.83±3.65	103.16±4.49	Normal Control
**1.48±0.17**	423.66±10.38	48.66±2.58	102.33±3.61	*E.* *angustifolia*
**6.21±0.76 ** ^a^	713.50±34.58 ^a^	631.33±14.76 ^a^	709.50±12.56 ^a^	CCl_4_
**4.14±0.63 ** ^c^	511.50±10.98 ^d^	253.83±13.51^d^	303.66±7.96 ^d^	*E. angustifolia* + CCl_4_
**4.23±0.69 ** ^c^	508.83±12.87 ^d^	252.50±15.52 ^d^	329.66±10.30 ^d^	Silymarin + CCl_4_

*Data is expressed as the mean ± SD.* ^a^ *p<0.001 compared with normal control;* ^b^ *p<0.05,* ^c^ *p<0.01, and* ^d^ *p<0.001 compared with CCl*_4_

### Effects of E. angustifolia extract administration on bilirubin, protein and serum albumin

CCl_4_ injection significantly elevated total and direct bilirubin levels (p<0.001) and decreased total protein and serum albumin levels (p<0.001). Pretreatment with *E. angustifolia* extract mitigated these effects (p<0.05), as shown in Table 3.

### Effects of E. angustifolia extract administration on lipid profile

CCl_4_ administration increased total cholesterol, triglycerides, and LDL-C levels, while decreasing HDL-C levels (p<0.001). Pretreatment with *E. angustifolia* extract attenuated these changes (p<0.01), as shown in Table 4.

### Effects of E. angustifolia extract administration on lipid peroxidation

**Figure 1:**
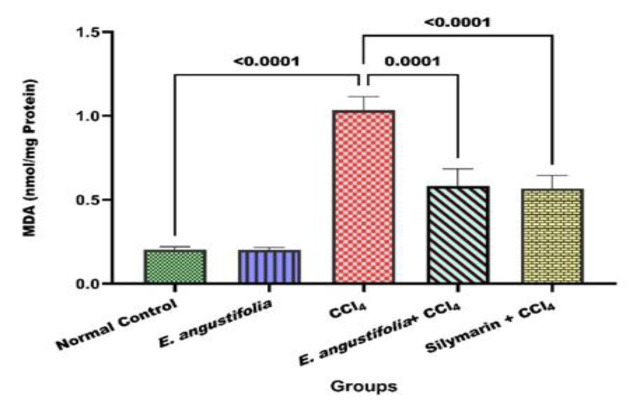
The effect of Elaeagnus angustifolia L. fruit extract on the level of lipid peroxidation. Data is expressed as the mean ± SD.

Figure 1 shows that CCl_4_ administration significantly increased MDA levels compared to the normal control group (p<0.001). Pretreatment with *E. angustifolia* extract reduced MDA levels significantly (p<0.001).

### Effects of E. angustifolia extract administration on total antioxidant capacity

**Figure 2:**
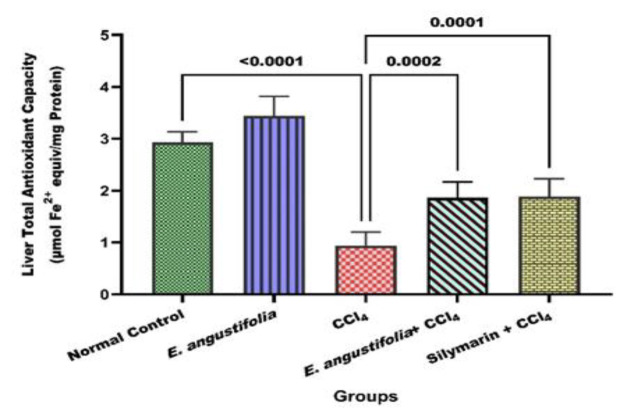
The effect of Elaeagnus angustifolia L. fruit extract on total antioxidant capacity. Data is expressed as the mean ± SD.

As shown in Figure 2, CCl_4_ significantly decreased total antioxidant capacity (p<0.001). Pretreatment with *E. angustifolia* extract reversed this decline, significantly increasing total antioxidant capacity in liver tissue samples (p<0.01).

**Table 3:** The effect of Elaeagnus angustifolia L. fruit extract on serum bilirubin, total protein, and albumin levels

Albumin (g/dl)	Total Protein (g/dl)	BilD (mg/dl)	BilT (mg/dl)	Groups
**3.18** **±0.17**	7.19±0.13	0.08±0.01	0.65±0.03	Normal Control
**3.23±0.19**	7.12±0.44	0.07±0.01	0.64±0.02	*E.* *angustifolia*
**2.36±0.24 ** ^a^	5.03±0.48 ^a^	0.32±0.03 ^a^	1.11±0.11^a^	CCl_4_
**2.91±0.11** ^d^	6.21±0.55 ^c^	0.21±0.03 ^d^	0.84±0.08 ^b^	*E. angustifolia* + CCl_4_
**2.88±0.11** ^d^	6.07±0.68 ^c^	0.21±0.02 ^d^	0.87±0.08 ^b^	Silymarin + CCl_4_

* Data is expressed as the mean ± SD.* ^a^ *p<0.001 compared with normal control;* ^b^ *p<0.05,* ^c^ *p<0.01, and* ^d^ *p<0.001 compared with CCl*_4_

**Table 4:** Effect of Elaeagnus angustifolia L. fruit extract on lipid profile

HDL (mg/dl)	LDL (mg/dl)	Triglyceride (mg/dl)	Cholesterol (mg/dl)	Groups
**39.41** **±1.50**	26.50±1.87	48.38±2.77	68.66±3.50	Normal Control
**38.81±2.19**	24.83±2.63	46.51±3.81	69.16±2.63	*E.* *angustifolia*
**24.96±1.92 ** ^a^	51.83±5.81 ^a^	191.51±6.58 ^a^	87.16±6.33 ^a^	CCl_4_
**31.83±2.74 ** ^d^	34.50±4.13 ^d^	142.50±4.84 ^d^	74.50±4.27 ^d^	*E. angustifolia* + CCl_4_
**30.53±2.63 ** ^c^	33.66±4.08 ^d^	131.33±5.98 ^d^	72.83±5.63 ^d^	Silymarin + CCl_4_

Data is expressed as the mean ± SD. ^a^p<0.001 compared with normal control; ^b^p<0.05, ^c^p<0.01, and ^d^p<0.001 compared with CCl_4_

### Effects of E. angustifolia extract administration on antioxidant enzymes

**Figure 3:**
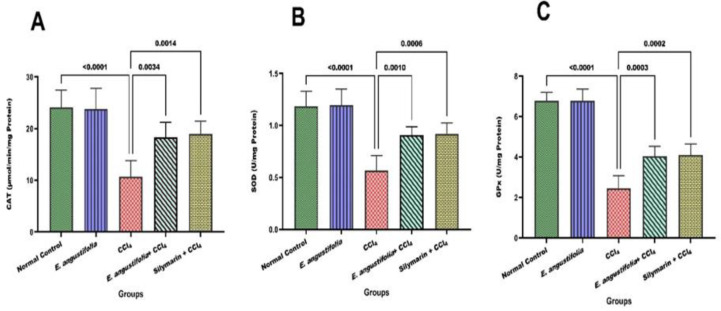
The effect of Elaeagnus angustifolia fruit extract on antioxidant enzymes activity. Data is expressed as the mean ± SD*. (A: Catalase, B: SOD, and C: GPx)*

A single CCl_4_ injection significantly reduced antioxidant enzyme (CAT, SOD, and GPx) activity (p<0.001). Pretreatment with *E. angustifolia* extract prevented this reduction significantly (p<0.05), as shown in Figure 3.

### Effects of E. angustifolia extract administration on inflammatory cytokines

**Figure 4:**
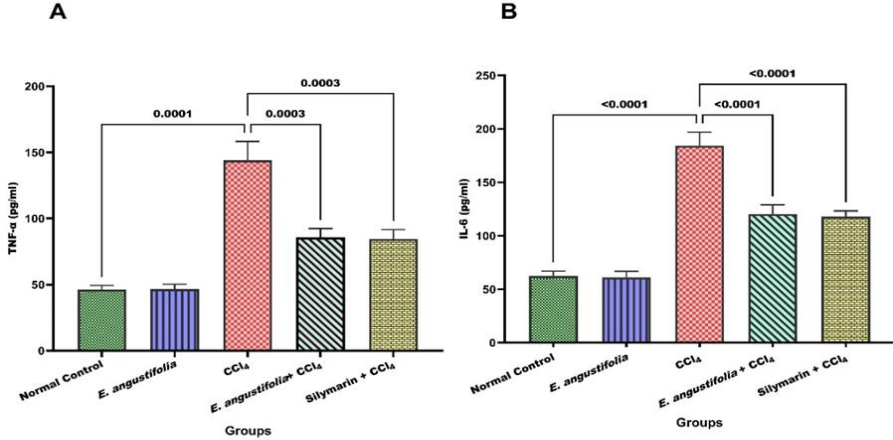
The effect of Elaeagnus angustifolia L. fruit extract on inflammatory cytokines. Data is expressed as the mean ± SD. (A: TNF-α and B: IL-6)

Serum TNF-α and IL-6 significantly increased with CCl_4_ injection (p<0.001). Pretreatment with *E. angustifolia* extract significantly prevented this increase, similar to silymarin (Figure 4). 

### Histopathological study

**Figure 5:**
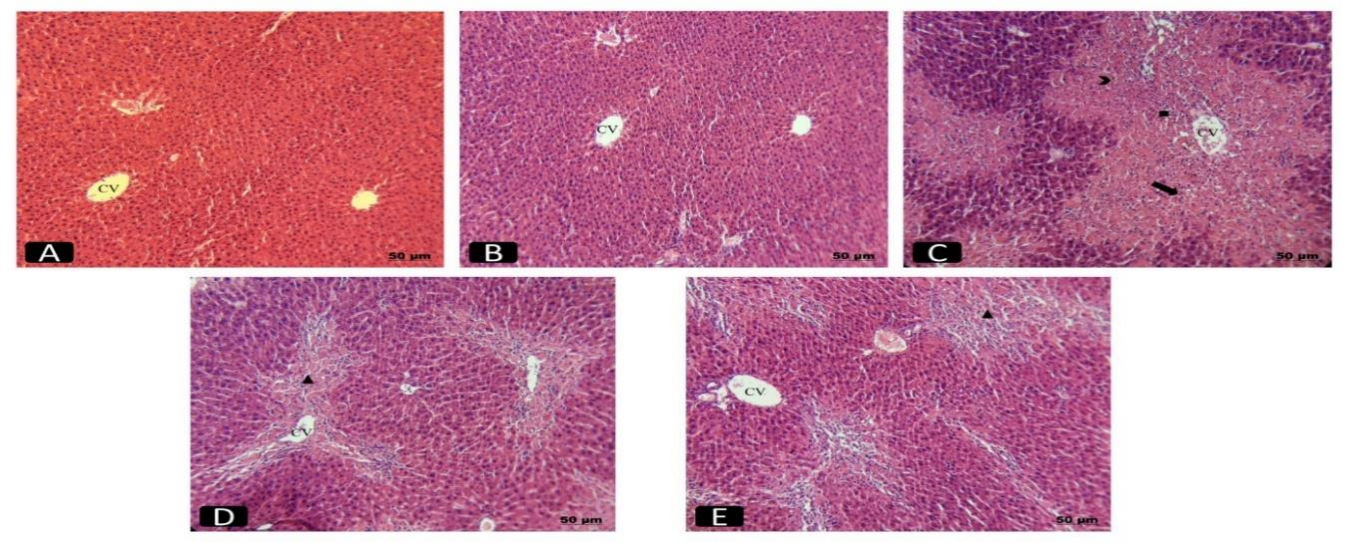
Microscopic images of liver tissue in different groups: A- Normal control group. B- E. angustifolia group. C- CCl4 group. D- E. angustifolia + CCl4 group. E- Silymarin + CCl4 group. CV Central vein. Necrosis. Irregular cell plates. Inflammation. Hepatocyte degeneration

Histopathological observations (Figure 5 and Table 5) confirmed the protective effect of E. angustifolia fruit extract against liver damage. CCl_4_ induced extensive changes in the lobules, such as fat accumulation, cellular vacuolation and necrosis, sinusoidal dilation and inflammatory cell infiltration, in the CCl_4_ group. However, the extract preserved hepatocyte structure and reduced necrosis and inflammation, similar to silymarin.

### Liver index

*The CCl*_4_ *-injured group showed a significant increase (p<0.05) in liver index. Pretreatment with E. angustifolia extract led to a significant decrease (p<0.05) in this index (* Table 6 *).*

**Table 5:** Histological injury score of the liver

Groups	Index							
	Injury of Score^ᴬ^							
	Hepatocyte degeneration	Sinusoidal dilation	Portal area dilation	Irregular cell plates	Necrosis	Inflammation	Fatty degeneration	Total scores
**Normal control**	0	0	0	0	0	0	0	0
***E. angustifolia***	0	0	0	0	0	0	0	0
**CCl**_4_	3	3	2	3	3	2	2	18
***E. angustifolia*** ** + CCl**_4_	2	1	1	2	2	1	1	10
**Silymarin + CCl**_4_	1	1	1	1	1	1	1	7

ᴬ The degree of hepatic damage was assessed by examining the livers under a microscope and assigning a score based on the following criteria: score 0=no signs of cell injury; 1= hepatocyte injury on less than 25% of the tissue; score 2= hepatocyte injury on 25–50% of the tissue; score 3= immense, but focal, hepatocyte injury; score 4= overall hepatocyte necrosis.

**Table 6:** The effect of Elaeagnus angustifolia L. fruit extract on liver index

Liver index (%)	Liver weight (gram)	Body weight on day 16 (gram)	Body weight on day 1 (gram)	Groups
**2.94** **±0.15**	7.26±0.25	247.33±5.68	207.66±5.00	Normal control
**2.98±0.14**	7.43±0.36	249.16±3.06	209.16±5.41	*E.* *angustifolia*
**4.64±0.18 ** ^a^	10.90±0.46 ^a^	243.66±6.08	210.33±6.18	CCl_4_
**3.61±0.09 ** ^d^	8.78±0.30 ^d^	243.33±7.31	209.66±6.43	*E. angustifolia* + CCl_4_
**3.55±0.18 ** ^d^	8.63±0.36 ^d^	242.83±5.77	208.16±6.79	Silymarin + CCl_4_

Data is expressed as the mean ± SD. ^a^ p<0.001 compared with normal control; ^b^ p<0.05, ^c^ p<0.01, and ^d^ p<0.001 compared with CCl_4_

## Discussion

This study evaluated *E. angustifolia* fruit extract for antioxidant and anti-inflammatory effects against CCl_4_-induced liver injury, demonstrating efficacy in reducing damage. Biochemical markers were used to assess oxidative damage.

The cell membrane, rich in fatty acids, is highly susceptible to free radical damage. Leakage of cell contents into the bloodstream indicates cellular and tissue damage severity, assessable by measuring specific molecules in the blood (Stark 2005). Assessing liver enzymes in serum is a common method to evaluate liver damage (Meng et al. 2020). Significantly (p<0.001) elevated levels of ALT, AST, ALP, and GGT indicate hepatocyte leakage due to CCl_4_-induced liver injury in rats compared to the normal group. Liver damage also led to decreased serum protein and albumin levels, altered lipid profiles, and changes in bilirubin levels similar to the findings of other researchers (Ebeid et al. 2015; Saleh Gazwi and Mahmoud 2019; Ullah et al. 2020). Pretreatment with *E. angustifolia* fruit extract, rich in phenolic and flavonoid content, remarkably (p<0.01) protected against liver damage, potentially through its antioxidant properties, and via neutralizing free radicals and preventing further hepatocyte damage. We also investigated the antioxidant markers in liver tissue to further elucidate the liver-protection effects of *E. angustifolia *fruit extract.

MDA, a marker of lipid peroxidation, was utilized to evaluate oxidative damage in the liver (Gaweł et al. 2004). CCl_4_-induced free radicals can induce lipid peroxidation, causing breakdown of PUFA (Polyunsaturated fatty acids) molecules and harmful compound production (Negre-Salvayre et al. 2010). Studies have demonstrated that CCl_4_ raises MDA levels in rat liver tissue (Chen et al. 2020; Eltahir et al. 2020). CCl_4_ notably (p<0.001) increased lipid peroxidation, but *E. angustifolia* fruit extract significantly (p<0.01) decreased MDA levels, suggesting protection against oxidative damage.

Further investigation should focus on the impact of *E. angustifolia* fruit extract on the antioxidant enzyme system in liver tissue. Cellular antioxidant enzymes neutralize radicals and protect cells. However, the efficacy of this protective system diminishes when the balance of antioxidants and free radicals within the cell is disrupted (Mohammadi Mahjoob et al. 2024; Tahavvori et al. 2023; Wei et al. 2021). Exposure to CCl_4_ is known to disrupt this system, leading to cellular dysfunction (Almatroodi et al. 2020). CCl_4_ caused a significant (p<0.001) decrease in antioxidant enzymes (CAT, SOD, and GPx) and pretreatment with the extract preserved enzyme activity, indicating its potential to enhance antioxidant defense against free radicals.

CCl_4_-induced liver damage also involves inflammation as characterized by increased TNF-α and IL-6 levels (Long et al. 2022). This study confirmed elevated cytokines due to inflammation, consistent with previous research (Jaime-Pérez et al. 2020; Long et al. 2022; Said et al. 2022). The *E. angustifolia* fruit extract was found to inhibit the elevation of TNF-α and IL-6 levels, indicating the anti-inflammatory properties of the extract's compounds.

Histological analysis of rat liver tissue confirmed CCl_4_-induced damage, showing necrosis, cell infiltration, fibrosis, and steatosis like previous studies (Eidi et al. 2013a). Pre-treatment with *E. angustifolia* fruit extract improved liver structure, consistent with biochemical results, suggesting its potential for liver protection and tissue regeneration. Multiple studies have confirmed the extract's ability to reduce inflammation. This is achieved through the inhibition of cyclooxygenase, highlighting its potential therapeutic benefits in inflammatory conditions (Hamidpour et al. 2017).

Finally, the liver index, an important marker for assessing the severity of liver damage, was utilized to demonstrate the protective effects of the extract (Wei et al. 2021). CCl_4_ significantly elevated the liver index, consistent with previous research. Pre-treatment with the extract effectively (p<0.01) mitigated this increase, similar to silymarin. These results underscore the potential of *E. angustifolia* fruit extract's antioxidant properties in protecting against severe liver damage induced by CCl_4_.

This study revealed that *E. angustifolia* fruit extract exhibits antioxidant effects and enhances liver function against CCl_4_-induced liver injury in rats. 

## Abbreviations

ALP Alkaline phosphatase

ALT Alanine Transaminase

AST Aspartate aminotransferase

CAT Catalase

CCl_4_ Carbon tetrachloride

DPPH 2,2-diphenyl-1-picrylhydrazyl

FRAP Ferric Reducing Antioxidant Power Assay

GGT Gamma glutamyl transferase

GPx Glutathione peroxidase

HDL-C High Density Lipoprotein Cholesterol

IC50 Half-maximal inhibitory concentration

IL-6 Interleukin 6

LDL-C Low Density Lipoprotein Cholesterol

MDA Malondialdehyde

PUFA Polyunsaturated fatty acids

ROS Reactive oxygen species

SOD Superoxide dismutase

TNF-α Tumor necrosis factor alpha
